# Factors and management techniques in odontogenic keratocysts: a systematic review

**DOI:** 10.1186/s40001-024-01854-z

**Published:** 2024-05-15

**Authors:** Mario Dioguardi, Cristian Quarta, Diego Sovereto, Giorgia Apollonia Caloro, Andrea Ballini, Riccardo Aiuto, Angelo Martella, Lorenzo Lo Muzio, Michele Di Cosola

**Affiliations:** 1 Department of Clinical and Experimental Medicine, University of Foggia, Via Rovelli 50, 71122 Foggia, Italy; 2 Unità Operativa Nefrologia e Dialisi, Presidio Ospedaliero Scorrano, ASL (Azienda Sanitaria Locale) Lecce, Via Giuseppina Delli Ponti, 73020 Scorrano, Italy; 3 Department of Biomedical, Surgical, and Dental Science, University of Milan, 20122 Milan, Italy; 4 DataLab, Department of Engineering for Innovation, University of Salento, Lecce, Italy

## Abstract

**Objectives:**

Odontogenic keratocysts exhibit frequent recurrence, distinctive histopathological traits, a tendency towards aggressive clinical behavior, and a potential linkage to the nevoid basal cell carcinoma syndrome. The aim of this systematic review is to compile insights concerning the control of this condition and assess the effectiveness of various treatment approaches in reducing the likelihood of recurrence.

**Materials and methods:**

The following systematic review adhered to the PRISMA guidelines. The systematic revision was registered on PROSPERO and  structured around the questions related to the population, intervention, control, outcome and study design (PICOS).

**Results:**

After conducting a search on the PubMed database, we initially identified 944 records. After using end-note software to remove duplicate entries, results totally with 462 distinct records. A thorough review of the titles and abstracts of these articles led to the selection of 50 papers for in-depth examination. Ultimately, following the application of our eligibility criteria, we incorporated 11 articles into our primary outcome analysis.

**Conclusion:**

Among the studies examined, the most common location for these lesions was found to be in the area of the mandibular ramus and the posterior region of the mandible. In cases where the exact location wasn’t specified, the mandible emerged as the predominant site. When we considered the characteristics of these lesions in studies that mentioned locularity, most were described as unilocular in two studies, while in two other studies, the prevalence of multilocular lesions was observed. Risk factors associated with keratocyst recurrence include younger patient age, the presence of multilocular lesions, larger lesion size, and a longer anteroposterior dimension. Certain treatment methods have demonstrated a lack of relapses. These include the use of 5-fluorouracil, marsupialization, enucleation with peripheral ostectomy or resection, enucleation and curettage, as well as resection without creating continuity defects. However, it is important to note that further research is essential. Prospective studies and randomized trials are needed to collect more comprehensive evidence regarding the effectiveness of various treatment approaches and follow-up protocols for managing odontogenic keratocysts.

**Clinical relevance:**

Odontogenic keratocysts still enter into differential diagnoses with other lesions that affect the jaw bones such as ameloblastama and other tumor forms, furthermore it is not free from recurrence, therefore the therapeutic approach to the lesion aimed at its elimination can influence both the possible recurrence and complications, knowledge of the surgical methods that offer the most predictable and clinically relevant result for the management of follow-up and recurrences.

## Introduction

The odontogenic keratocyst (OKC) is a developmental cyst that originates from remnants of the dental lamina within the jawbones [1]. Several studies have reported a preference for males [1–3], with an incidence peak around the third decade [4] and a nearly equal distribution in other decades, with another small peak between 50 and 70 years of age [1]. It can occur in any area of the jawbones but is most commonly found in the mandible, with a particular preference for the mandibular angle extending to the mandibular ramus [4].

Diagnosis of OKC is typically  radiological. Radiographs commonly reveal well-defined radiolucent areas with  rounded or scalloped margins that are well demarcated; these areas can present as either multilocular or unilocular [5].

In the 2022 classification, OKC remains classified as a cyst; molecular studies have detected frequent mutations in the tumor suppressor gene PTCH1, a gene that activates the SHH pathway, leading to aberrant epithelial proliferation [1], sparking debates on whether OKC is a cyst or a cystic neoplasm. It was labeled as a keratocystic odontogenic tumor in 2005 [5], thus considered a cystic neoplasm, and later reclassified as a cyst in the 2017 classification [1].

Keratocysts are characterized by a high recurrence rate, specific histological features, aggressive clinical behavior, and can be associated with the nevoid basal cell carcinoma syndrome [6].

The mechanism of recurrence was proposed by Brannon [7] in 1976, suggesting it was due to three different mechanisms:

- Incomplete removal of the cyst,
- Growth of new keratocysts from satellite cysts,
- Development of a new keratocyst in the area adjacent to the site of the primary keratocyst, interpreted as recurrence.

Odontogenic keratocysts can be treated with various surgical methods, which can be divided into conservative approaches and invasive approaches or a combination thereof [8]; in the literature, enucleation, marsupialization, resection, and the use of adjunct therapies such as Carnoy’s solution and cryotherapy are reported [1, 4, 9].

Despite many studies in the literature examining several therapeutic approaches in managing this lesion, it is still not clear which method provides lower recurrence rates without causing significant morbidity [10]; the purpose of this systematic review is to gather information on the management of this lesion and evaluate which treatment method results in fewer recurrences.

## Materials and methods

The following systematic review adhered to the PRISMA (Preferred Reporting Items for Systematic review and Meta-Analysis) protocol guidelines [11].

The systematic revision was registered on PROSPERO with number of: CRD42023480051.

The study was structured around the questions related to the population, intervention, control, outcome and study design (PICOS):

Population (P): individuals with non-syndromic or syndromic odontogenic keratocyst (initial cases) diagnosed histologically;

Intervention (I): surgical interventions for patients with odontogenic keratocystic, such as enucleation, enucleation coupled with curettage, enucleation with additional therapeutic measures (such as Carnoy's solution application, cryotherapy), marsupialization or decompression, with or without subsequent cystectomy and adjunctive therapy, and resection;

Control (C): not applicable;

Outcome (O): recurrence of KOT (Keratocystic Odontogenic Tumor) associated with distinct surgical treatments and characteristics of the keratocysts analyzed;

Study design (S): prospective randomized controlled clinical trials, controlled clinical investigations (either prospective or retrospective), and case series that explored and compared the diverse surgical approaches concerning recurrence over a suitable follow-up period (minimum of 1 year).

The formulation of the PICOS question can be summarized as follows: “What characteristics do the odontogenic keratocysts analyzed in the studies have? Which surgeries had the least recurrences during the follow-up?”.

Following the initial selection phase of records identified in various databases, potentially eligible articles were qualitatively assessed. This assessment aimed to investigate which surgical treatment was the most reliable in giving the least number of recurrences.

### Eligibility criteria

This text discusses the process of selecting research articles for a study related to the recurrence of KOT associated with distinct surgical interventions, such as enucleation, with or without curettage and additional therapeutic measures, marsupialization or decompression, with or without subsequent cystectomy and adjunctive therapy, and resection.

The process involved initially identifying potentially eligible articles based on their abstracts. These articles were then subjected to a thorough examination of their full content to determine their suitability for both qualitative and quantitative analyses.

The criteria for including articles in the full-text analysis were studies relating to KOT treatments in which the number of recurrences and the general characteristics of the lesions are reported.

The exclusion criteria were applied to exclude the following types of studies:

- Studies involving animals or conducted in a laboratory setting (in vitro)
- Letters to the editor
- Articles that did not adequately specify the type of surgical method used
- Studies with an inadequate follow-up period (less than 1 year)
- Clinical studies conducted more than 30 years ago (only studies from the last 30 years were included because classifications and surgical and therapeutic techniques have been constantly changing and improving, with generally earlier diagnoses and more suitable treatments with lower recurrence rates. Therefore, to avoid increasing the heterogeneity of the included studies and to prevent bias in the aggregated treatment results, the reviewers collectively decided to include only studies from 1989 onwards)
- Review articles

### Research methodology

Studies have been identified through bibliographic research on electronic databases.

The literature search was conducted on the search engines “PubMed”. The search on the providers was conducted between 02.09.2023 and 12.09.2023, and the last search for a partial update of the literature was conducted on 18.09.2023.

The following search terms were used on PubMed: “KOT” AND “Recurrence” (37 records), “odontogenic keratocyst marsupialization” (285 records), “odontogenic keratocyst enucleation” (622 records).

### Screening methodology

The selection criteria and their combinations for searching were established prior to the record identification stage through mutual consensus between the two reviewers  (M.D. and M.D.C.) responsible for choosing potentially eligible articles. Following this, the records acquired were then assessed separately by the two independent reviewers, with a third reviewer  (A.B.) serving as an decision-maker in cases of uncertainty.

The screening process involved evaluating the titles and abstracts of articles, and in cases where there was uncertainty, a more in-depth examination of the article's content was conducted to remove records that were not relevant to the topics under review.

## Results

**Fig. 1:**
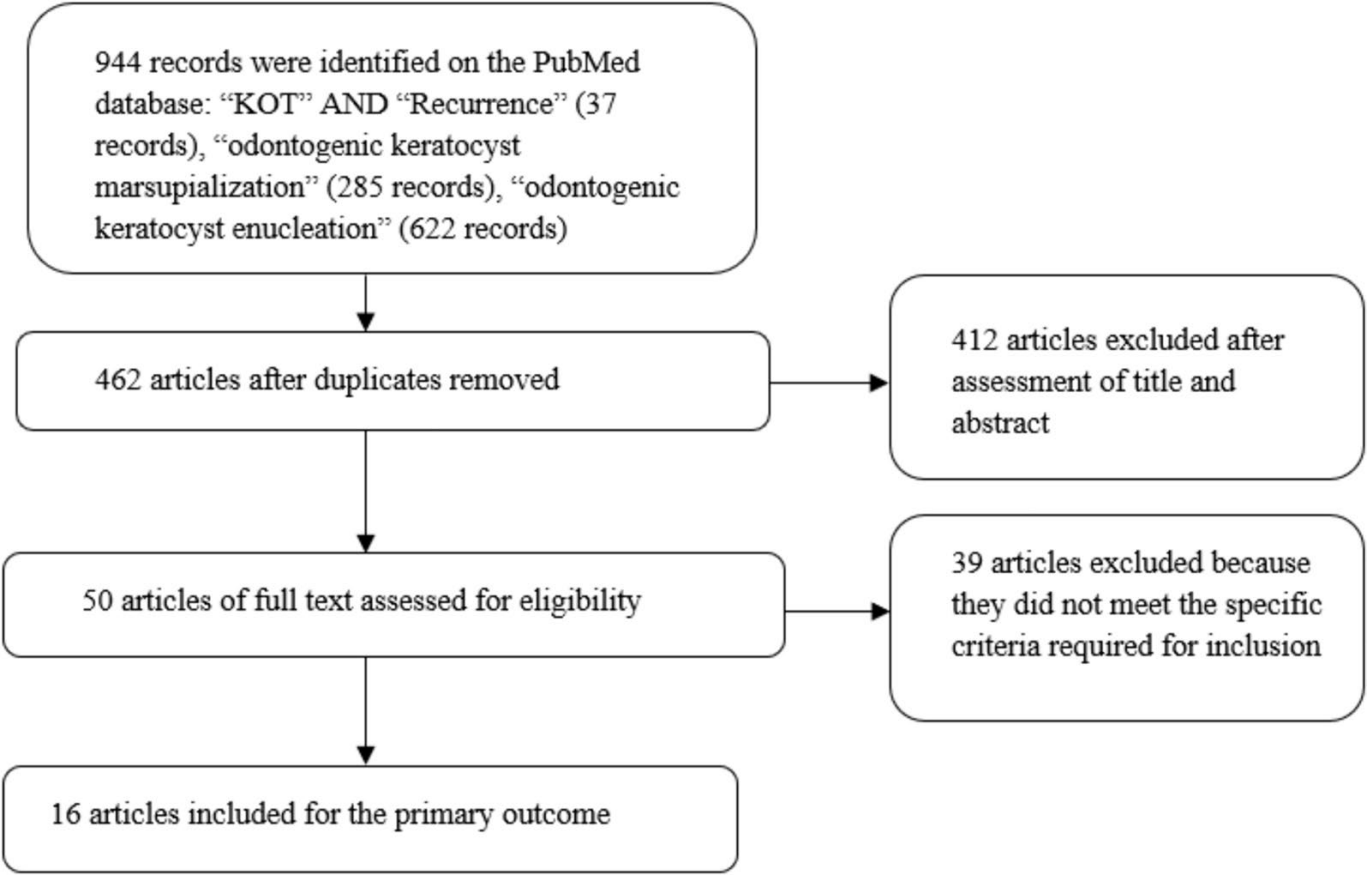
Flowchart of the different phases of the systematic review

Following a search in the PubMed database, 944 records were initially located. Subsequently, after applying end-note software to eliminate duplications, 462 unique records remained. Upon reviewing the titles and abstracts of these articles, after this initial screening, a total of 50 articles were selected for a thorough examination of their full text by two reviewers. From these 50 articles, the ones that met the criteria for qualitative analysis for the outcome were identified. Finally, applying the eligibility criteria, we included 16 articles for the primary outcome analysis (Fig. 1).

### Study characteristics and data extraction

The included studies for the quantitative analysis were: Maurette et al. [12]; Nakamura et al. [13]; Bataineh and al Qudah [14]; Leung et al. [15]; Kolokythas et al. [9]; Berge et al. [16]; Pogrel and Jordan, [17]; Tabrizi et al. [18]; Zecha et al. [19]; Moellmann et al. [20]; Caminiti et al.[21], Stoelinga [4]; Dammer et al. [2]; Marker et.al. [22]; August et al.[23]; Brøndum and Jensen [24].

The extracted data included the journal (author, data, and reference); study design; number of patients (males/females); number of lesions; number of lesions associated with basal cell naevus syndrome (BCNS); mean age (range); site where the lesions were diagnosed; locularity (multilocular or unilocular); type of treatment; mean follow-up.

Finally, for each study, the number of relapses relating to each treatment was observed.

The data extracted are shown in Table 1 and 2.

**Table 1:** Characteristics of odontogenic keratocysts, M male, F female, NS not available

First author, date, journal	Study design	N. of patients (M/F)	N. of lesions	Association with BCNS (n. of lesion)	Mean age (range)	Site (n. of patients)	Locularity
Maurette, 2006, [12]	Retrospective	28 (9/19)	30	None	30 (13–69)	Angle and Ramus of Mandible (16), Other side of the jaws (12)	NS
Nakamura, 2002, [13]	Retrospective	24 (14/10)	28	5	35,1 (11–70)	Angle and Ramus of Mandible (14), Anterior molar region (12) Anterior maxilla (2)	Unilocular (21) Multilocular (7)
Bataineh and al Qudah, 1998, [14]	Retrospective	31 (20/11)	31	NS	34 (15–67)	Ramus and angular region (23) Body of mandible (8)	Unilocular (12); multilocular (19)
Leung, 2016 [15]	Retrospective	105 (54/51)	105	None	37,1 (10–83)	Posterior maxilla (20) Anterior maxilla (2) Posterior mandible (79) Anterior mandible (4)	Unilocular (44); multilocular (61)
Kolokythas, 2007 [9]	Retrospective	22 (10/12)	22	None	46,8 (18–90)	Right mandible (7) Left mandible (9) Anterior maxilla (2) Posterior maxilla (2) Right maxilla (2) Maxilla (1)	NS
Berge, 2016, [16]	Retrospective	92 (59/33)	92	None	48 (-)	mandible posterior (60) mandible anterior (7) maxilla anterior (10) maxilla posterior (15)	NS
Pogrel, 2004, [17],	Retrospective	10 (6/4)	10	NS	NS (11–64)	NS	NS
Tabrizi, 2012, [18]	Case series	13 (8/5)	13	None	22,4 (16–31)	Mandible (13)	NS
Zecha, 2010, [19]	Retrospective	68 (43/25)	68	None	39,5 (12–79)	Maxilla anterior (6) Maxilla posterior (10) Mandible anterior (3) Mandible posterior (35) Ramus of mandible (14)	NS
Moellmann, 2023, [20]	Retrospective	111 (74/37)	111	None	NS (12–90)	Mandible (102) Maxilla (9)	NS
Caminiti, 2021, [21]	Retrospective	70 (36/34)	70	None	47	Mandible (22) Maxilla (12)	Unilocular: 24 Multilocular: 9
					38	Mandible (31) Maxilla (5)	Unilocular: 24 Multilocular: 12
August et al., 2003 [23]	Retrospective	14 (6/8)	14	None	32 (9–62)	Mandible (10) Maxilla (4)	Unilocular: 5 Multilocular: 9
Stoelinga, 2001 [4]	Prospective	80 (39\41)	82	None	10–89	Mandible (68) Maxilla (14)	Unilocular: 40 Scalloped 17 Multilobular 18 Multilocular: 7
Dammer et al., 1997 [2]	Retrospective	38 (29\9)	42 (52)^a^	None	37 (14–65)	Mandible (26) Maxilla (16)	Unilocular: 28, Multilocular: 10
Marker et.al.,1997, [22]	Prospective study	23 (14/9)	23	None	47 (10–87)	Mandible (20) Maxilla (3)	NS
Brøndum and Jensen 1991, [24]	Retrospective	44 (22\22)	44\54^b^	None	45 (9–87)	Mandible (44) Maxilla (10)	Unilocular: 26, Multilocular:18

^**a**^52 keratocysts if we include a single patient with Gorlin goltz who has 10 odontogenic keratocysts, 5 in the mandible and 5 in the maxilla

^b^The text is not clear, sometimes it reports 44 cases of keratocysts and other times it reports it as the number of patients included and then identifies 54 keratocysts

**Table 2:** Treatments, number of recurrence and follow-up

First author, date, journal	Treatment (n. of patients)	N. of recurrence	Follow-up
Maurette, 2006, [12]	Decompression and curettage of the remaining lesion (20)	4	24.9 months
	Enucleation and curettage (10)	0	
Nakamura, 2002 [13]	Marsupialization only (5)	0	79.2 months
	Marsupialization followed by enucleation and curettage (23)	6	
Bataineh and al Qudah, 1998 [14]	Resection without continuity defects (31)	0	2–8 years
Leung, 2016 [15]	Enucleation and the application of carnoy’s solution (105)	12	86.6 months
Kolokythas, 2007, [9]	Enucleation with peripheral ostectomy or resection (11)	0	1,5–9 years
	decompression followed by enucleation and peripheral ostectomy (11)	2	1,5–3 years
Berge, 2016, [16]	Enucleation (70)	23	66 months
	Marsupialization with subsequent enucleation (22)	4	
Pogrel, 2004, [17]	Marsupialization (10)	0	2.8 years
Tabrizi, 2012, [18]	Marsupialization (13)	0	60 months
Zecha, 2010 [19]	Enucleation (58)	12	65.1 months
	Maruspialization (10)	4	
Moellmann, 2023 [20]	Cistostomy (25)	1	41.9 months
	Cistectomy (70)	20	
	Cistectomy + carnoy’s solution (5)	14	
	Cistectomy and curettage (8)	5	
	Partial resection of the jaw (3)	1	
Caminiti, 2021, [21]	5-fluorouracil after enucleation and ostectomy (34)	0	30 months
	Modified carnoy’s solution after enucleation and ostectomy (36)	9	35 months
August et al., 2003 [23]	Decompression and residual cystectomy	0	33.6 months
Stoelinga, 2001 [4]	Carnoy’s solution after enucleation	6	1–25 years
Dammer et al., 1997 [2]	Cistostomy or carnoy’s solution	3	
Marker et.al.,1997, [22]	Decompression and later cystectomy	2	1–19 years
Brøndum and Jensen, 1991 [24]	Decompression and later cystectomy	8	9 years

### Risk of bias

The risk of bias was assessed using the Newcastle–Ottawa Scale (NOS) for cohort studies, assigning a value from 0 to 3 for each item, the assessment of the risk of bias was assessed by the first reviewer, and was deemed acceptable for all included studies, details are shown in Table 3

**Table 3:** Risk of bias, NOS

First author, date, journal	Selection					Comparability	Outcome			Total scor
	Study design	Representativeness of cohort	Selection of non-exposed cohort	Ascertainment of exposure	Outcome of interest	Comparability of cohorts	Assessment of outcome	Adequate duration of followup	Adequate followup of cohort	
Maurette, 2006, [12]	Retrospective	2	2	2	2	1	1	1	1	12
Nakamura, 2002 [13]	Retrospective	1	1	1	1	1	2	2	2	11
Bataineh and al Qudah, 1998, [14]	Retrospective	1	2	1	1	1	2	2	2	12
Leung, 2016 [15]	Retrospective	2	3	2	2	1	2	2	2	16
Kolokythas, 2007 [9]	Retrospective	1	1	1	2	1	*2	*2	2	12
Berge, 2016 [16]	Retrospective	2	2	2	1	1	*2	*2	2	14
Pogrel, 2004 [17]	Retrospective	1	1	1	1	2	1	2	1	10
Tabrizi, 2012 [18]	Retrospective\ case series?	1	1	1	*2	*1	1	1	1	9
Zecha, 2010, [19]	Retrospective	2	2	2	1	1	1	1	1	11
Moellman, 2023 [20]	Retrospective	2	2	2	2	2	2	2	2	16
Caminiti, 2021 [21]	Retrospective	2	1	1	1	1	1	2	2	11
August et al., 2003 [23]	Retrospective	2	1	1	1	1	1	2	2	11
Stoelinga, 2001 [4]	Prospective	2	2	2	1	1	1	2	2	13
Dammer et al., 1997 [2]	Retrospective	1	2	1	1	1	2	2	1	11
Marker et.al.,1997, [22]	Prospective	1	1	1	1	1	2	2	2	11
Brøndum and Jensen 1991, [24]	Retrospective	1	1	1	1	1	2	2	2	11

## Discussion

The articles included in this review analyze different types of keratocyst treatment and lesion characteristics.

Among the first to coin the term 'odontogenic keratocyst' was Philipsen in 1956, who, in a literature review, proposed the term 'odontogenic keratocyst' for all odontogenic cysts that exhibit epithelial keratinization [25].

The terminology, as adopted by Pindborg in 1962 and 1963 and also used by Toller in 1967, replaced the term ‘primordial cyst’ with ‘odontogenic keratocyst’, identifying 33 odontogenic keratocysts (study not included in this review) [26–29]

One of the early retrospective studies conducted on odontogenic keratocysts was performed by Pindborg, who retrospectively identified 26 keratinized cysts out of a total of 791 odontogenic cysts in 1962 [27].

The odontogenic keratocysts are often described in literature as benign cysts occurring within the bones, and they exhibit a propensity for infiltrative and aggressive growth patterns. These cysts make up an estimated 2–21.8% of all cysts affecting the jaw [24, 25]. Moreover, there is a potential association between these cysts and genetic mutations, notably linked to nevoid basal cell carcinoma syndrome (NBCCS), a condition characterized by the presence of multiple OKCs in the jaw region [26]; this is also found in one of the articles included in this review [13], while in others the association was not specified [14, 17] or there was no association at all [9, 12, 15, 16, 18–21]; many of these studies have placed the correlation with this syndrome in the exclusion criteria, as in the patients who are affected by it the probability that these cysts will reappear is high, and therefore it would be difficult to distinguish a recurrent event from the appearance of a new cyst [21]

These cysts are notorious for their tendency to grow aggressively in their immediate prossimity and for having a notably high rate of recurrence. Several contributing factors underpin this recurrence, including the use of inadequate treatment methods, incomplete elimination of the cyst, a high rate of cell division (mitotic index) within the cyst's epithelial cells, a larger cyst size, and the specific location of the cyst. The latter factor becomes especially problematic if it is challenging to access surgically [25, 27]. Although they exhibit hostile conduct, OKC generally induce limited bone enlargement as they tend to proliferate within the intramedullary region, effectively growing within the bone [30].

Substantial lesions marked by substantial cortical plate erosion and engagement with neighboring structures may not produce symptoms in individuals, resulting in a delayed diagnosis [31].

The most frequent location of the lesions in the studies analyzed is at the level of the mandibular ramus and in the posterior mandible [12–16, 19], and where the precise localization of the lesions is not specified, the mandible is the most frequent site [9, 18, 20, 21]. In the studies in which locularity is specified among the characteristics of the lesions, the majority of the lesions were unilocular in two studies [13, 21], while in two other studies the quantity of multilocular lesions was greater [14, 15]. Younger patient age, multilocularity of the lesion, larger size, and longer anteroposterior dimension of the keratocyst have been identified as risk factors for keratocyst recurrence [15].

The treatments that have not had relapses are that with 5-fluorouracil [21], marsupialization [13, 17, 18], enucleation with peripheral ostectomy or resection [9], enucleation and curettage [12], and resection without continuity defects [14].

Decompression has been studied in 5 articles [9, 12, 22–24]; this method has the advantage of having minimal surgical morbidity and reduced risk to anatomical structures associated with the lesion, such as developing nerves or teeth [22]. Decompression and marsupialization techniques involve creating a communication between the cyst and the oral cavity, relieving pressure and allowing cyst shrinkage and bone apposition [12]. Clinical and radiographic resolution of OKCs after marsupialization is relatively rapid, typically within 19 months [17]. In studies where marsupialization alone was used for treatment, there were no relapses in two studies [17, 18], while Zecha et al. [19] found four cases of relapse in ten patients treated with marsupialization.

Decompression and marsupialization are non-invasive treatment options for keratocysts, but require patient cooperation, including regular irrigation and follow-up [17, 18].

Topical 5-fluorouracil is known for its antiproliferative effects on keratocystic epithelium and satellite cysts; furthermore, its use has some advantages, such as technical ease and the lack of neurotoxicity [21] and, in the only study of this review in which it were used in the treatment, there were no relapses [21].

Other treatment modalities used to reduce keratocyst recurrence are resection of the affected maxillary segment and enucleation with additional treatments such as curettage or ostectomy [9, 14], which in these studies have not given recurrences, which, as regards resection, is a similar result to other studies in the literature [4, 8, 32]. However, despite the remarkably high success rate of this approach, resection is not widely embraced as a standard procedure, primarily due to concerns regarding its aggressiveness and associated postoperative complications, including morbidity [33]. Enucleation, often combined with curettage (the process of scraping the walls of the lesion cavity) or ostectomy (the surgical removal of bone tissue), is commonly used to treat keratocysts; although a more conservative treatment than resection, the effectiveness of this modality may be limited in cases where vital structures, such as the exposed inferior alveolar nerve, are at risk or when there is a perforation of the bony wall exposing the overlying mucosal tissue [15].

Carnoy’s solution was used in three studies [15, 20, 21] and of these studies one used the modified Carnoy’s solution [21]. The FDA avoid the use of Carnoy's solution containing chloroform in the United States, leading to the adoption of a modified formula. However, the modified formula has been found to have a higher relapse rate, suggesting the potential role that traditional Carnoy’s solution may have in treatment [34].

## Conclusion

There are risk factors associated with the recurrence of odontogenic keratocyst, such as age, multilocularity, lesion size and radiographic characteristics.

The various surgical techniques used to treat keratocysts have potential benefits, including preservation of jaw function, reduction of the potential for recurrence, and eradication of the cystic lesion.

Marsupialization or decompression are advantageous conservative treatment options that aim to minimize surgical invasiveness while effectively managing keratocysts.

Long-term follow-up and monitoring of patients treated for these lesions is important to detect recurrence early.

There is a need for further research, prospective studies and randomized trials to gather more evidence on the effectiveness of different treatment methods and follow-up protocols for odontogenic keratocysts.

## Data Availability

All data generated or analyzed during this study are included in this published article.
